# Crystal structure of 2,5-dimethyl-3-(3-methyl­phenyl­sulfon­yl)-1-benzo­furan

**DOI:** 10.1107/S1600536814019369

**Published:** 2014-08-30

**Authors:** Hong Dae Choi, Uk Lee

**Affiliations:** a Department of Chemistry, Dongeui University, San 24 Kaya-dong, Busanjin-gu, Busan 614-714, Republic of Korea; b Department of Chemistry, Pukyong National University, 599-1 Daeyeon 3-dong, Nam-gu, Busan 608-737, Republic of Korea

**Keywords:** crystal structure, benzo­furan, 3-methyl­phen­yl, C—H⋯π hydrogen bonds, π–π inter­actions

## Abstract

In the title compound, C_17_H_16_O_3_S, the dihedral angle between the plane of the benzo­furan ring system [r.m.s. deviation = 0.010 (1) Å] and that of the 3-methyl­phenyl ring is 79.09 (5)°. Intra­molecular C—H⋯O hydrogen bonds are observed. In the crystal, mol­ecules are connected into a chain along the *c*-axis direction by two different pairs of inversion-generated inter­actions: C—H⋯π hydrogen bonds between the methyl groups and the benzene rings of the 3-methyl­phenyl fragments and π–π inter­actions between the benzene and furan rings of neighbouring mol­ecules [centroid–centroid distance = 3.673 (2) Å].

## Related literature   

For the pharmacological properties of compounds containing a benzo­furan moiety, see: Aslam *et al.* (2009 ▶); Galal *et al.* (2009 ▶); Howlett *et al.* (1999 ▶); Khan *et al.* (2005 ▶); Ono *et al.* (2002 ▶). For natural products with a benzo­furan ring, see: Akgul & Anil (2003 ▶); Soekamto *et al.* (2003 ▶). For the synthesis of the starting material, 2,5-dimethyl-3-(3-methyl­phenyl­sulfan­yl)-1-benzo­furan, see: Choi *et al.* (1999 ▶). For a related structure, see: Choi *et al.* (2013 ▶).
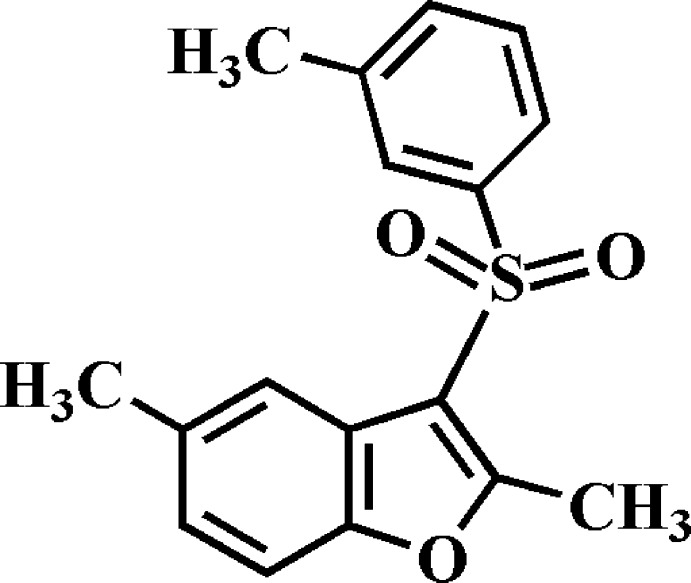


## Experimental   

### Crystal data  

C_17_H_16_O_3_S
*M* *_r_* = 300.36
Monoclinic, 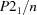

*a* = 9.8769 (2) Å
*b* = 11.3190 (2) Å
*c* = 13.1320 (2) Å
β = 101.951 (1)°
*V* = 1436.29 (4) Å^3^
*Z* = 4
Mo *K*α radiation
μ = 0.23 mm^−1^
*T* = 173 K
0.33 × 0.29 × 0.23 mm

### Data collection  

Bruker SMART APEXII CCD diffractometer
Absorption correction: multi-scan (*SADABS*; Bruker, 2009 ▶) *T*_min_ = 0.927, *T*_max_ = 0.947
13810 measured reflections
3589 independent reflections
2861 reflections with *I* > 2σ(*I*)
*R*_int_ = 0.035

### Refinement  

*R*[*F* ^2^ > 2σ(*F* ^2^)] = 0.045
*wR*(*F* ^2^) = 0.128
*S* = 1.08
3589 reflections
193 parameters
H-atom parameters constrained
Δρ_max_ = 0.35 e Å^−3^
Δρ_min_ = −0.39 e Å^−3^

### 

Data collection: *APEX2* (Bruker, 2009 ▶); cell refinement: *SAINT* (Bruker, 2009 ▶); data reduction: *SAINT*; program(s) used to solve structure: *SHELXS97* (Sheldrick, 2008 ▶); program(s) used to refine structure: *SHELXL97* (Sheldrick, 2008 ▶); molecular graphics: *ORTEP-3 for Windows* (Farrugia, 2012 ▶) and *DIAMOND* (Brandenburg, 1998 ▶); software used to prepare material for publication: *SHELXL97*.

## Supplementary Material

Crystal structure: contains datablock(s) I. DOI: 10.1107/S1600536814019369/fy2118sup1.cif

Structure factors: contains datablock(s) I. DOI: 10.1107/S1600536814019369/fy2118Isup2.hkl

Supporting information file. DOI: 10.1107/S1600536814019369/fy2118Isup3.cml

**Fig. 1.**
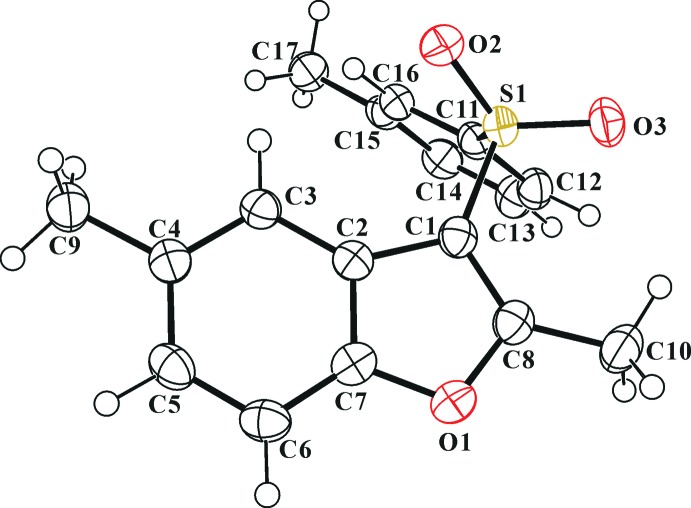
The molecular structure of the title compound with the atom numbering scheme. Displacement ellipsoids are drawn at the 50% probability level. H atoms are presented as small spheres of arbitrary radius.

**Fig. 2.**
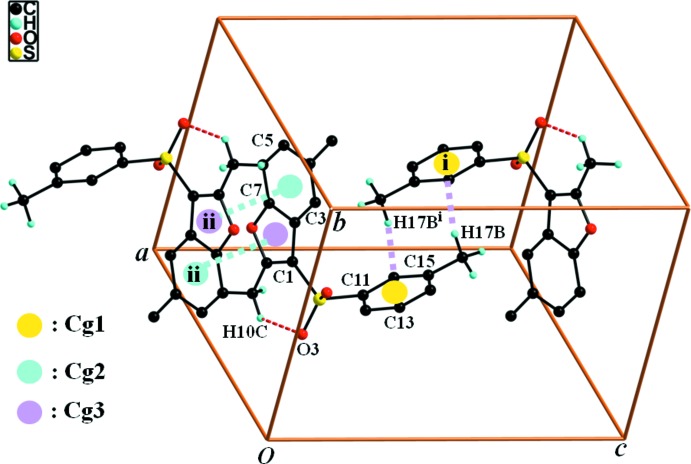
A view of the C–H···O, C–H···π and π–π interactions (dotted lines) in the crystal structure of the title compound. H atoms non-participating in hydrogen-bonding were omitted for clarity. [Symmetry codes: (i) - x + 1, - y + 1, - z + 1; (ii) - x + 1, - y + 1, - z.]

. DOI: 10.1107/S1600536814019369/fy2118fig1.tif
The mol­ecular structure of the title compound with the atom numbering scheme. Displacement ellipsoids are drawn at the 50% probability level. H atoms are presented as small spheres of arbitrary radius.

x y z x y z . DOI: 10.1107/S1600536814019369/fy2118fig2.tif
A view of the C–H⋯O, C–H⋯π and π–π inter­actions (dotted lines) in the crystal structure of the title compound. H atoms non-participating in hydrogen-bonding were omitted for clarity. [Symmetry codes: (i) − *x* + 1, − *y* + 1, − *z* + 1; (ii) − *x* + 1, − *y* + 1, − *z*.]

CCDC reference: 1021292

Additional supporting information:  crystallographic information; 3D view; checkCIF report

## Figures and Tables

**Table 1:** Hydrogen-bond geometry (Å, °) *Cg*1 is the centroid of the C11–C16 3-methyl­phenyl ring.

*D*—H⋯*A*	*D*—H	H⋯*A*	*D*⋯*A*	*D*—H⋯*A*
C10—H10*C*⋯O3	0.98	2.34	3.078 (3)	131
C17—H17*B*⋯*Cg*1^i^	0.98	2.88	3.478 (2)	121

Symmetry code: (i) 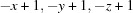.

## supplementary crystallographic information

### S1. Comment

Molecules containing a benzofuran ring show interesting pharmacological properties such as antibacterial and antifungal, antitumor and antiviral, antimicrobial activities (Aslam *et al.* 2009, Galal *et al.*, 2009, Khan *et al.*, 2005), and they are potential inhibitors of β-amyloid aggregation (Howlett *et al.*, 1999, Ono *et al.*, 2002). These benzofuran compounds are widely occurring in nature (Akgul & Anil, 2003; Soekamto *et al.*, 2003). As a part of our ongoing program of 3-arylsulfonyl-2,5-dimethyl-1-benzofuran derivatives containing 4-methylphenylsulfonyl substituent in 3-position (Choi *et al.*, 2013), we report herein on the crystal structure of the title compound.

In the title molecule (Fig. 1), the benzofuran unit is essentially planar, with a mean deviation of 0.010 (1) Å from the least-squares plane defined by the nine constituent atoms. The 3-methylphenyl ring is essentially planar, with a mean deviation of 0.005 (1) Å from the least-squares plane defined by the six constituent atoms. The dihedral angle formed by the benzofuran ring system and the 3-methylphenyl ring is 79.09 (5)°. In the crystal structure (Fig. 2), molecules are linked along the *c*-axis direction by two different inversion-generated pairs of C–H···π hydrogen bonds (Table 1, Cg1 is the centroid of the C11-C16 3-methylphenyl ring), and π–π interactions between the benzene and furan rings of neighbouring molecules, with a Cg2···Cg3^ii^ distance of 3.673 (2) Å and an interplanar distance of 3.570 (2) Å resulting in a slippage of 0.864 (2) Å (Cg2 and Cg3 are the centroids of the C2-C7 benzene ring and the C1/C2/C7/O1/C8 furan ring, respectively). In addition, intramolecular C–H···O hydrogen bonds (Table 1) are observed .

### S2. Experimental

The starting material 2,5-dimethyl-3-(3-methylphenylsulfanyl)-1-benzofuran was prepared by literature method (Choi *et al.* 1999). 3-Chloroperoxybenzoic acid (77%, 515 mg, 2.3 mmol) was added in small portions to a stirred solution of 2,5-dimethyl-3-(3-methylphenylsulfanyl)-1-benzofuran (295 mg, 1.1 mmol) in dichloromethane (30 mL) at 273 K. After being stirred at room temperature for 8h, the mixture was washed with saturated sodium bicarbonate solution (2 X 20 mL) and the organic layer was separated, dried over magnesium sulfate, filtered and concentrated at reduced pressure. The residue was purified by column chromatography (hexane-ethyl acetate, 4:1 *v*/*v*) to afford the title compound as a colorless solid [yield 73% (241 mg); m.p. 378-379 K; *R*_f_ = 0.43 (hexane-ethyl acetate, 4:1 *v*/*v*)]. Single crystals suitable for X-ray diffraction were prepared by slow evaporation of a solution of the title compound (28 mg) in acetone (15 mL) at room temperature.

### S3. Refinement

All H atoms were positioned geometrically and refined using a riding model, with C–H = 0.95 Å for aryl and 0.98 Å for methyl H atoms, *U*_iso_ (H) = 1.2*U*_eq_ (C) for aryl and 1.5*U*_eq_ (C) for methyl H atoms.The positions of methyl hydrogens were optimized using the SHELXL-97's command AFIX 137 (Sheldrick, 2008).

### Crystal data

**Table:**

C_17_H_16_O_3_S	*F*(000) = 632
*M**_r_* = 300.36	*D*_x_ = 1.389 Mg m^−^^3^
Monoclinic, *P*2_1_/*n*	Melting point = 378–379 K
Hall symbol: -P 2yn	Mo *K*α radiation, λ = 0.71073 Å
*a* = 9.8769 (2) Å	Cell parameters from 3458 reflections
*b* = 11.3190 (2) Å	θ = 2.4–25.7°
*c* = 13.1320 (2) Å	µ = 0.23 mm^−^^1^
β = 101.951 (1)°	*T* = 173 K
*V* = 1436.29 (4) Å^3^	Block, colourless
*Z* = 4	0.33 × 0.29 × 0.23 mm

### Data collection

**Table:**

Bruker SMART APEXII CCD diffractometer	3589 independent reflections
Radiation source: rotating anode	2861 reflections with *I* > 2σ(*I*)
Graphite multilayer monochromator	*R*_int_ = 0.035
Detector resolution: 10.0 pixels mm^-1^	θ_max_ = 28.4°, θ_min_ = 2.4°
φ and ω scans	*h* = −13→12
Absorption correction: multi-scan (*SADABS*; Bruker, 2009)	*k* = −14→15
*T*_min_ = 0.927, *T*_max_ = 0.947	*l* = −17→17
13810 measured reflections	

### Refinement

**Table:**

Refinement on *F*^2^	Primary atom site location: structure-invariant direct methods
Least-squares matrix: full	Secondary atom site location: difference Fourier map
*R*[*F*^2^ > 2σ(*F*^2^)] = 0.045	Hydrogen site location: difference Fourier map
*wR*(*F*^2^) = 0.128	H-atom parameters constrained
*S* = 1.08	*w* = 1/[σ^2^(*F*_o_^2^) + (0.0639*P*)^2^ + 0.4013*P*] where *P* = (*F*_o_^2^ + 2*F*_c_^2^)/3
3589 reflections	(Δ/σ)_max_ < 0.001
193 parameters	Δρ_max_ = 0.35 e Å^−^^3^
0 restraints	Δρ_min_ = −0.39 e Å^−^^3^

### Special details

**Table:**

Experimental. ^1^H NMR (δ p.p.m., CDCl3, 400 Hz): 7.77-7.83 (m, 2H), 7.67 (s, 1H), 7.34-7.41 (m, 2H), 7.29 (d, J = 8.56 Hz, 1H), 7.10 (dd, J = 8.56 Hz and 1.72 Hz, 1H), 2.78 (s, 3H), 2.45 (s, 3H), 2.40 (s, 3H).
Geometry. All esds (except the esd in the dihedral angle between two l.s. planes) are estimated using the full covariance matrix. The cell esds are taken into account individually in the estimation of esds in distances, angles and torsion angles; correlations between esds in cell parameters are only used when they are defined by crystal symmetry. An approximate (isotropic) treatment of cell esds is used for estimating esds involving l.s. planes.
Refinement. Refinement of F^2^ against ALL reflections. The weighted R-factor wR and goodness of fit S are based on F^2^, conventional R-factors R are based on F, with F set to zero for negative F^2^. The threshold expression of F^2^ > 2sigma(F^2^) is used only for calculating R-factors(gt) etc. and is not relevant to the choice of reflections for refinement. R-factors based on F^2^ are statistically about twice as large as those based on F, and R- factors based on ALL data will be even larger.

### Fractional atomic coordinates and isotropic or equivalent isotropic displacement parameters (Å^2^)

**Table:**

	*x*	*y*	*z*	*U*_iso_*/*U*_eq_	
S1	0.29608 (4)	0.36272 (4)	0.17488 (3)	0.02967 (14)	
O1	0.66012 (13)	0.37182 (11)	0.09943 (10)	0.0345 (3)	
O2	0.20801 (13)	0.46348 (11)	0.14867 (10)	0.0366 (3)	
O3	0.24998 (14)	0.24951 (11)	0.13176 (10)	0.0384 (3)	
C1	0.45457 (18)	0.39515 (16)	0.14324 (12)	0.0287 (4)	
C2	0.52013 (17)	0.51022 (15)	0.14998 (12)	0.0273 (4)	
C3	0.48710 (18)	0.62452 (15)	0.17608 (13)	0.0297 (4)	
H3	0.4006	0.6408	0.1940	0.036*	
C4	0.58250 (19)	0.71390 (16)	0.17537 (13)	0.0306 (4)	
C5	0.70909 (19)	0.68878 (18)	0.14708 (14)	0.0356 (4)	
H5	0.7734	0.7511	0.1468	0.043*	
C6	0.74318 (19)	0.57618 (17)	0.11965 (14)	0.0361 (4)	
H6	0.8287	0.5597	0.1003	0.043*	
C7	0.64635 (19)	0.48979 (16)	0.12196 (13)	0.0306 (4)	
C8	0.54295 (19)	0.31645 (16)	0.11373 (13)	0.0315 (4)	
C9	0.5504 (2)	0.83842 (17)	0.20414 (15)	0.0385 (4)	
H9A	0.4861	0.8749	0.1458	0.058*	
H9B	0.6362	0.8845	0.2199	0.058*	
H9C	0.5082	0.8367	0.2654	0.058*	
C10	0.5398 (2)	0.18729 (17)	0.09586 (16)	0.0410 (5)	
H10A	0.6052	0.1485	0.1523	0.062*	
H10B	0.5660	0.1704	0.0293	0.062*	
H10C	0.4463	0.1574	0.0941	0.062*	
C11	0.33565 (18)	0.34750 (15)	0.31193 (14)	0.0293 (4)	
C12	0.3862 (2)	0.24005 (16)	0.35446 (15)	0.0350 (4)	
H12	0.3997	0.1762	0.3107	0.042*	
C13	0.4166 (2)	0.22735 (18)	0.46169 (15)	0.0405 (5)	
H13	0.4527	0.1549	0.4922	0.049*	
C14	0.3941 (2)	0.32033 (18)	0.52383 (15)	0.0383 (4)	
H14	0.4147	0.3104	0.5973	0.046*	
C15	0.34268 (18)	0.42744 (16)	0.48270 (14)	0.0321 (4)	
C16	0.31430 (18)	0.44131 (16)	0.37446 (14)	0.0306 (4)	
H16	0.2806	0.5146	0.3441	0.037*	
C17	0.3154 (2)	0.52658 (18)	0.55131 (15)	0.0387 (4)	
H17A	0.2158	0.5323	0.5488	0.058*	
H17B	0.3486	0.6009	0.5270	0.058*	
H17C	0.3641	0.5114	0.6230	0.058*	

### Atomic displacement parameters (Å^2^)

**Table:**

	*U*^11^	*U*^22^	*U*^33^	*U*^12^	*U*^13^	*U*^23^
S1	0.0298 (2)	0.0263 (2)	0.0322 (2)	−0.00279 (17)	0.00464 (17)	−0.00376 (16)
O1	0.0356 (7)	0.0348 (7)	0.0345 (6)	0.0044 (5)	0.0105 (5)	−0.0018 (5)
O2	0.0326 (7)	0.0328 (7)	0.0437 (7)	0.0038 (5)	0.0062 (6)	0.0015 (6)
O3	0.0415 (8)	0.0308 (7)	0.0403 (7)	−0.0082 (6)	0.0027 (6)	−0.0085 (6)
C1	0.0305 (9)	0.0286 (9)	0.0259 (8)	−0.0005 (7)	0.0036 (7)	−0.0013 (6)
C2	0.0276 (8)	0.0302 (9)	0.0230 (7)	0.0000 (7)	0.0026 (6)	0.0015 (6)
C3	0.0278 (9)	0.0307 (9)	0.0301 (8)	−0.0001 (7)	0.0050 (7)	−0.0002 (7)
C4	0.0318 (9)	0.0306 (9)	0.0272 (8)	−0.0003 (7)	0.0008 (7)	0.0040 (7)
C5	0.0327 (9)	0.0371 (11)	0.0367 (9)	−0.0059 (8)	0.0066 (7)	0.0085 (8)
C6	0.0315 (9)	0.0431 (11)	0.0355 (9)	0.0018 (8)	0.0109 (7)	0.0062 (8)
C7	0.0340 (9)	0.0333 (10)	0.0247 (8)	0.0047 (8)	0.0064 (7)	0.0024 (7)
C8	0.0349 (9)	0.0317 (10)	0.0268 (8)	0.0014 (8)	0.0035 (7)	−0.0018 (7)
C9	0.0408 (11)	0.0325 (10)	0.0415 (10)	−0.0034 (8)	0.0066 (8)	0.0004 (8)
C10	0.0458 (11)	0.0337 (11)	0.0427 (10)	0.0065 (9)	0.0075 (9)	−0.0075 (8)
C11	0.0277 (9)	0.0279 (9)	0.0330 (8)	−0.0063 (7)	0.0078 (7)	−0.0021 (7)
C12	0.0384 (10)	0.0289 (10)	0.0391 (9)	−0.0042 (8)	0.0112 (8)	−0.0014 (8)
C13	0.0472 (12)	0.0335 (11)	0.0416 (10)	−0.0008 (9)	0.0115 (9)	0.0056 (8)
C14	0.0392 (10)	0.0424 (11)	0.0348 (9)	−0.0034 (9)	0.0110 (8)	0.0037 (8)
C15	0.0263 (8)	0.0358 (10)	0.0357 (9)	−0.0053 (7)	0.0098 (7)	−0.0051 (8)
C16	0.0282 (9)	0.0282 (9)	0.0359 (9)	−0.0032 (7)	0.0079 (7)	−0.0016 (7)
C17	0.0403 (11)	0.0411 (11)	0.0363 (9)	−0.0004 (9)	0.0115 (8)	−0.0036 (8)

### Geometric parameters (Å, º)

**Table:**

S1—O2	1.4324 (13)	C9—H9B	0.9800
S1—O3	1.4364 (13)	C9—H9C	0.9800
S1—C1	1.7399 (18)	C10—H10A	0.9800
S1—C11	1.7693 (18)	C10—H10B	0.9800
O1—C8	1.363 (2)	C10—H10C	0.9800
O1—C7	1.380 (2)	C11—C16	1.385 (2)
C1—C8	1.359 (2)	C11—C12	1.387 (3)
C1—C2	1.449 (2)	C12—C13	1.385 (3)
C2—C7	1.390 (2)	C12—H12	0.9500
C2—C3	1.394 (2)	C13—C14	1.378 (3)
C3—C4	1.384 (2)	C13—H13	0.9500
C3—H3	0.9500	C14—C15	1.381 (3)
C4—C5	1.405 (3)	C14—H14	0.9500
C4—C9	1.510 (3)	C15—C16	1.399 (2)
C5—C6	1.385 (3)	C15—C17	1.498 (3)
C5—H5	0.9500	C16—H16	0.9500
C6—C7	1.372 (3)	C17—H17A	0.9800
C6—H6	0.9500	C17—H17B	0.9800
C8—C10	1.480 (3)	C17—H17C	0.9800
C9—H9A	0.9800		
			
O2—S1—O3	119.24 (8)	C4—C9—H9C	109.5
O2—S1—C1	107.84 (8)	H9A—C9—H9C	109.5
O3—S1—C1	108.75 (9)	H9B—C9—H9C	109.5
O2—S1—C11	108.50 (8)	C8—C10—H10A	109.5
O3—S1—C11	107.42 (8)	C8—C10—H10B	109.5
C1—S1—C11	104.08 (8)	H10A—C10—H10B	109.5
C8—O1—C7	106.93 (14)	C8—C10—H10C	109.5
C8—C1—C2	107.54 (16)	H10A—C10—H10C	109.5
C8—C1—S1	126.36 (15)	H10B—C10—H10C	109.5
C2—C1—S1	125.99 (13)	C16—C11—C12	121.31 (17)
C7—C2—C3	119.19 (17)	C16—C11—S1	119.99 (14)
C7—C2—C1	104.37 (15)	C12—C11—S1	118.70 (13)
C3—C2—C1	136.45 (16)	C13—C12—C11	118.97 (17)
C4—C3—C2	118.84 (17)	C13—C12—H12	120.5
C4—C3—H3	120.6	C11—C12—H12	120.5
C2—C3—H3	120.6	C14—C13—C12	119.69 (19)
C3—C4—C5	119.88 (17)	C14—C13—H13	120.2
C3—C4—C9	120.16 (17)	C12—C13—H13	120.2
C5—C4—C9	119.95 (17)	C13—C14—C15	122.04 (18)
C6—C5—C4	122.18 (17)	C13—C14—H14	119.0
C6—C5—H5	118.9	C15—C14—H14	119.0
C4—C5—H5	118.9	C14—C15—C16	118.42 (17)
C7—C6—C5	116.23 (17)	C14—C15—C17	121.33 (17)
C7—C6—H6	121.9	C16—C15—C17	120.24 (17)
C5—C6—H6	121.9	C11—C16—C15	119.54 (17)
C6—C7—O1	125.76 (17)	C11—C16—H16	120.2
C6—C7—C2	123.67 (18)	C15—C16—H16	120.2
O1—C7—C2	110.56 (16)	C15—C17—H17A	109.5
C1—C8—O1	110.60 (16)	C15—C17—H17B	109.5
C1—C8—C10	134.37 (18)	H17A—C17—H17B	109.5
O1—C8—C10	115.02 (16)	C15—C17—H17C	109.5
C4—C9—H9A	109.5	H17A—C17—H17C	109.5
C4—C9—H9B	109.5	H17B—C17—H17C	109.5
H9A—C9—H9B	109.5		
			
O2—S1—C1—C8	−150.31 (15)	C1—C2—C7—O1	−0.07 (18)
O3—S1—C1—C8	−19.69 (18)	C2—C1—C8—O1	−0.81 (19)
C11—S1—C1—C8	94.58 (16)	S1—C1—C8—O1	−177.28 (12)
O2—S1—C1—C2	33.86 (17)	C2—C1—C8—C10	178.18 (19)
O3—S1—C1—C2	164.48 (14)	S1—C1—C8—C10	1.7 (3)
C11—S1—C1—C2	−81.25 (16)	C7—O1—C8—C1	0.77 (18)
C8—C1—C2—C7	0.53 (18)	C7—O1—C8—C10	−178.44 (15)
S1—C1—C2—C7	177.01 (12)	O2—S1—C11—C16	−16.89 (17)
C8—C1—C2—C3	−179.26 (19)	O3—S1—C11—C16	−147.04 (14)
S1—C1—C2—C3	−2.8 (3)	C1—S1—C11—C16	97.75 (15)
C7—C2—C3—C4	−1.3 (2)	O2—S1—C11—C12	162.26 (14)
C1—C2—C3—C4	178.44 (18)	O3—S1—C11—C12	32.11 (17)
C2—C3—C4—C5	1.0 (3)	C1—S1—C11—C12	−83.10 (15)
C2—C3—C4—C9	−179.47 (15)	C16—C11—C12—C13	−0.5 (3)
C3—C4—C5—C6	−0.2 (3)	S1—C11—C12—C13	−179.62 (15)
C9—C4—C5—C6	−179.74 (17)	C11—C12—C13—C14	1.1 (3)
C4—C5—C6—C7	−0.2 (3)	C12—C13—C14—C15	−0.5 (3)
C5—C6—C7—O1	−178.79 (16)	C13—C14—C15—C16	−0.7 (3)
C5—C6—C7—C2	−0.1 (3)	C13—C14—C15—C17	178.39 (19)
C8—O1—C7—C6	178.39 (17)	C12—C11—C16—C15	−0.7 (3)
C8—O1—C7—C2	−0.41 (18)	S1—C11—C16—C15	178.42 (13)
C3—C2—C7—C6	0.9 (3)	C14—C15—C16—C11	1.3 (3)
C1—C2—C7—C6	−178.90 (16)	C17—C15—C16—C11	−177.82 (16)
C3—C2—C7—O1	179.76 (14)		

### Hydrogen-bond geometry (Å, º)

*Cg*1 is the centroid of the C11–C16 3-methylphenyl ring.

**Table:**

*D*—H···*A*	*D*—H	H···*A*	*D*···*A*	*D*—H···*A*
C10—H10*C*···O3	0.98	2.34	3.078 (3)	131
C17—H17*B*···*Cg*1^i^	0.98	2.88	3.478 (2)	121

Symmetry code: (i) −*x*+1, −*y*+1, −*z*+1.
