# Supplementary figures and images for: Crystal structure of 2,5-dimethyl-3-(3-methyl­phenyl­sulfon­yl)-1-benzo­furan
Source: Acta Crystallogr Sect E Struct Rep Online. 2014 Aug 30;70(Pt 9):o1073–4. doi: 10.1107/S1600536814019369 (PMC4186096; doi:10.1107/S1600536814019369)

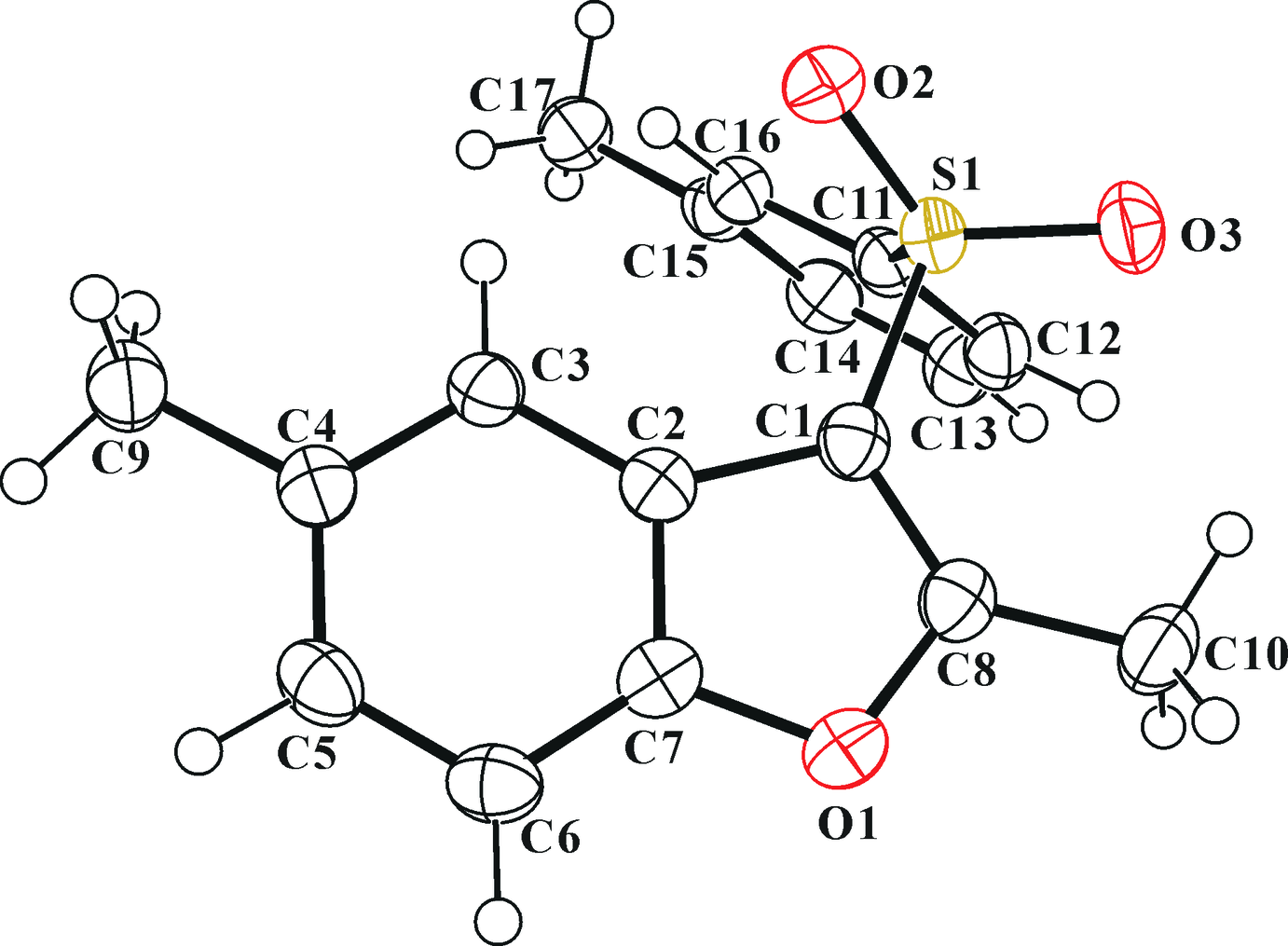

Supplement: Supplementary file 4 [file e-70-o1073-fig1.tif]

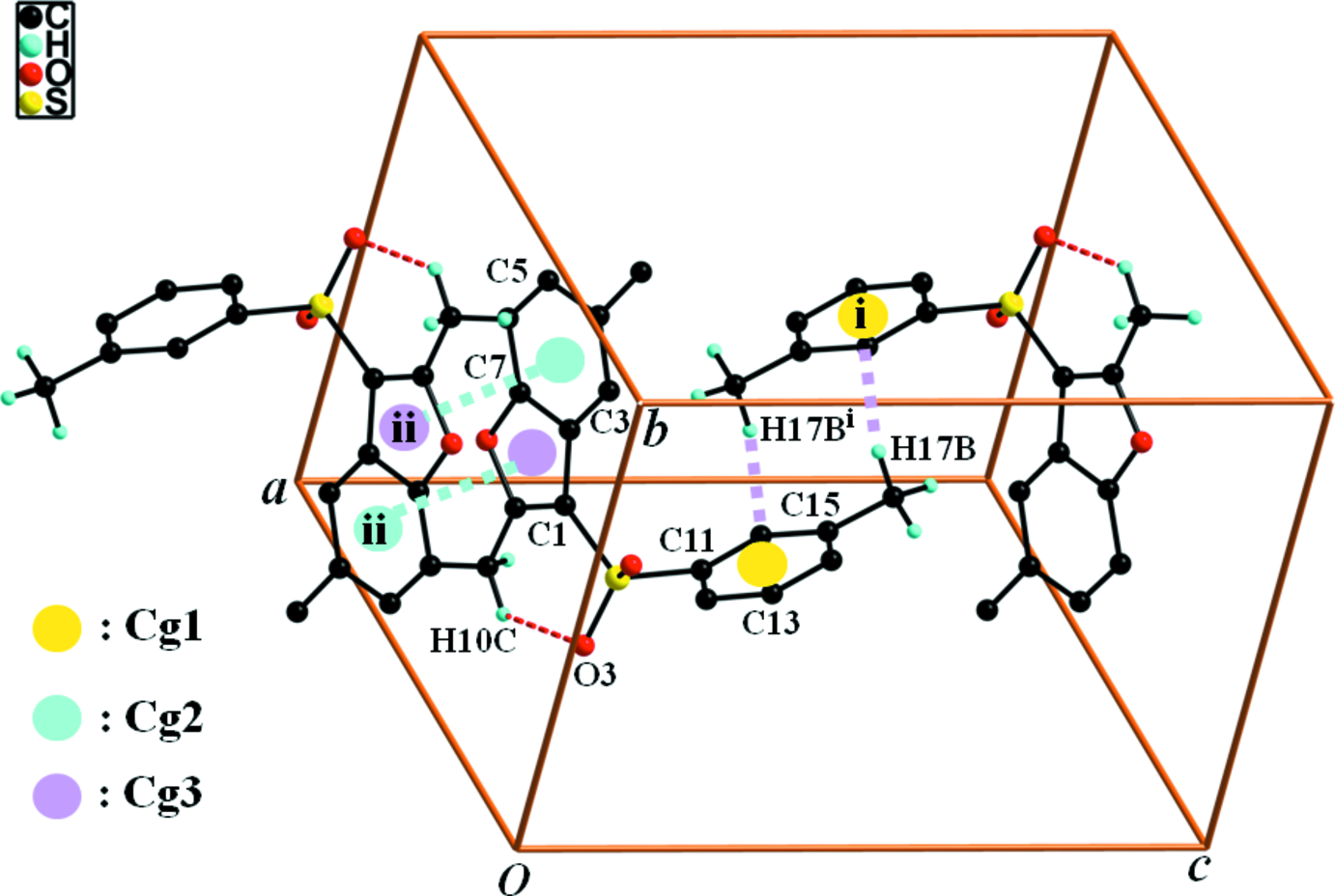

Supplement: Supplementary file 5 [file e-70-o1073-fig2.tif]
